# (Dis)continuation of the oral contraceptive pill: A focus group approach in the Netherlands^☆^

**DOI:** 10.1016/j.heliyon.2023.e19405

**Published:** 2023-08-23

**Authors:** R.S. Otte, V.T.M. Thissen, B.C. Mulder

**Affiliations:** Strategic Communication Group, Wageningen University & Research, P.O. BOX 8130, 6700, EW Wageningen, the Netherlands

**Keywords:** Oral contraception, (dis)continuation, Focus groups, Side effects, Communication, Responsibility

## Abstract

**Objective(s):**

The oral contraceptive pill (OCP) is the most used contraceptive worldwide; nevertheless, discontinuation rates are relatively high. While advantages of the OCP, like pregnancy prevention and planned parenthood, result in autonomy and independence, side effects and other aspects play a role in the decision to (dis)continue usage. Informed and deliberate decision-making is of importance to prevent adverse effects in health and quality of life. Therefore, the present study aimed to explore how women in the Netherlands decide to (dis)continue the OCP.

**Study design:**

This paper reports a qualitative study using four focus groups, conducted online or face-to-face, with 20 women of different educational background aged between 18 and 23. The topic guide was based on relevant theory and literature, and focused on personal beliefs, experiences and social interactions about the OCP. The study is reported according to SRQR guidelines.

**Results:**

The main themes that were found the influence decision-making were effects, side effects, towards alternatives, interpersonal communication with peers, interpersonal communication with the general practitioner, and online information seeking. Feeling responsible emerged as a relevant additional theme, embedded in the decision-making process, and influencing other themes. Some educational differences emerged.

**Conclusion(s):**

The decision to start the OCP is a highly normalized process in the Netherlands. However, women who feel a strong sense of responsibility for pregnancy prevention may be less open to considering alternative contraception methods. Results imply that women can be better supported in the decision-making process by providing balanced information on effects and side effects of a wide range of contraceptives, as well as restoring the balance in both sexual partner's responsibility for contraception. Implications for practice and suggestions for further research on the concept of responsibility, its influence, and underlying mechanisms are provided.

## Teaser

**Implications** Reasons to start using the OCP include managing menstrual pain, cycle control and pregnancy prevention. Over time, increasing awareness of physical (e.g., weight gain) and mental (e.g., mood swings) side effects may result in reconsidering the OCP. Interactions with peers and GP strongly influence the decision process.

## 1 Introduction

With approximately 151 million users, the oral contraceptive pill (OCP) is among the most widely used contraception methods worldwide [1]. The OCP enables women to regulate their periods, prevent pregnancy, and plan parenthood. These advantages have proven paramount for supporting women's autonomy and independence [2]. However, large population-based studies into OCP's show that 30%–60% of women discontinue using the OCP in 6–12 months [[3], [4], [5]].

In regard to OCP use, side effects are a major cause of discontinuation. Discontinuation, in this context, refers to either stopping OCP use altogether as well as switching to another contraception method. There are several concerns and dissatisfactions associated with the OCP. It has been reported that OCP's interact with vascular, endocrine and neurological body systems, thereby increasing the risk and severity of a stroke [6]. Furthermore, OCP use has been linked to an increased risk of breast cancer [7], and women experience side effects such as weight gain, impaired cognitive functioning, mood swings and irregular bleeding. All of these factors contribute to concerns and dissatisfaction with the OCP [8], which can ultimately lead to discontinuation [[9], [10], [11]]. Additionally, practical reasons such as running out of stock, difficulties obtaining the OCP, costs, and forgetfulness in taking it also contribute to discontinuation [[10], [11], [12]].

Understanding reasons for (dis)continuation is crucial for educating and supporting women in making optimal decisions regarding their contraceptive choices. Without making deliberate and informed decisions about continuing or discontinuing the OCP, women may, unwittingly, be at risk of decreasing their health and quality of life due to the unintended effects of (dis)continuation.

Although aforementioned pros and cons of OCP use have been examined, most studies focus on contraception decisions in general, rather than specifically on OCP decision-making. For instance, in the study of Simmons et al. [13], semi-structured interviews were used to develop a theory of how contraceptive decision-making occurs over time, identifying different phases in a person's contraceptive journey and five main areas of decisional influence within these phases. According to Sato et al. [14], both discontinuation rates and reasons of discontinuation seems to vary by the type of contraception method, highlighting the relevance of specifically investigating OCP use. Second, many studies fail to capture women's own experiences, while additionally their views are often categorised in medical terms rather than their own language [15]. Third, in Western societies where the OCP has become highly normalized, young women may experience influences from peers and health care providers encouraging them to start using the OCP [[16], [17], [18], [19]]. These social influences manifest as perceived norms, aligning with theory and literature that demonstrate behavior is strongly influenced by the actions and/or approval of significant others [20]. Specifically in the Netherlands, the use of the OCP is common and widely accepted. The OCP is available by prescription by general practitioners and is commonly used by women as a reliable method of birth control, often starting in their early teens. Women can conveniently obtain the oral contraceptive pill from community pharmacies for resupply without needing to renew the prescription for each dispensing [21]. It is worth noting that not all the countries have the same arrangements in place, which allow women to obtain repeat supplies from pharmacies without a prescription [22].

The Netherlands has high rate of contraceptive use. In 2017, 94% of young women and 92% of young men (aged 12–25 years) reported to have use any contraceptive during their first intercourse. The median age of OCP users in the Netherlands is 25–29 years [23]. In 2011, the Dutch College of General Practitioners adopted a new standard for contraception, which emphasized the importance of providing comprehensive information about a wide range of contraceptive methods to enable individuals to make informed decisions based on their personal preferences. Nevertheless, fixed combinations of estrogen and levonorgestrel (a second-generation progesterone) are the first choice in the Dutch guidelines on hormonal contraception and are therefore prescribed for the majority of women [24]. The costs of contraceptives (excluding condoms) for adolescents up to the age of 21 is covered by the Dutch basic insurance program.

Considering the high use of OCPs in the Netherlands, there is a need for research to understand the factors influencing women's decisions to (dis)continue their OCP use. Despite the widespread availability and acceptance of oral contraceptives, individual experiences and motivations may vary significantly and the reasons while some women continue with OCP use while others discontinue remain unclear. It is essential to investigate the unique factors at play in the Dutch setting, influenced by cultural attitudes, healthcare accessibility and social norms. It is of added value to investigate this within the context of a focus group study, as it provides an opportunity to capture the social aspect of decision-making. By engaging women in a group setting, the dynamics and interactions between participants can shed light on the influence of social norms, peer experiences, and shared perceptions related to oral contraceptive use. Therefore, the aim of the present study is to examine how women in the Netherlands decide to (dis)continue OCP use by exploring how decisions regarding the OCP are affected by their personal experiences, as well as social interactions with important others. This knowledge will provide valuable insights for improved contraceptive choices and tailored support that aligns with the specific needs and preferences of women in the Netherlands as well as other countries which share certain characteristics in terms of culture, social structure and healthcare provision, like Belgium, Germany, Sweden and Denmark.

## 2 Methods

### 2.1 Design

The research design employed in this study was a focus group study, as focus groups are particularly useful for exploring participants’ beliefs, feelings, and the construction of those beliefs and feelings based on personal experiences and interactions with others [25]. In June 2020, four focus groups were conducted, each lasting 1.5–2 h, with a total of 20 women between the ages of 18 and 23. Most of the participating women were in a relationship, with a small minority being mothers in focus group 1 and focus group 2. Some women were dating, but not in a committed relationship. Among the 20 women participating, 10 women were current OCP users, while the other 10 women were former OCP users (see Table 1 for an overview of participant characteristics).

**Table 1:** Participants Characteristics across four focus groups.

	Focus group 1	Focus group 2	Focus group 3	Focus group 4
Number of participants	5	4	6	5
Age range	18–21	18–23	21–23	21–23
Educational level	(preparatory)	Intermediate	Higher	University
	Intermediate	vocational	vocational	
	vocational	education	education	
	education			
Form	Online	Online	Online	Real life
Current OCP users	3	2	3	2
Former OCP users	2	2	3	3

All focus groups were moderated by the second author VT (female). Considering the conflicting evidence regarding the influence of socioeconomic factors on contraceptive discontinuation [10], it was meaningful to include participants with different educational backgrounds. Higher-educated participants (focus groups 3 and 4), were recruited via convenience and snowball sampling through the second author's network, utilizing a short video message distributed via WhatsApp messenger service. While the author knew some of the participants beforehand, not all were acquainted. Lower to middle educated women were not directly accessible; thus, participants for focus group 1 and 2 were recruited through Facebook, by posting the video message in groups with young adult women.

Due to measures implemented to curb the COVID-19 pandemic, organizing face-to-face ‘offline’ focus groups was challenging. As a result, only focus group 4 was offline at a mutually agreed location, while focus groups 1 to 3 were conducted via a WhatsApp group created specifically for this purpose. Therefore, all interactions in these groups occurred via text messages. After each focus group discussion, the conversations were saved in a Word document and the WhatsApp group was deleted. Recordings of the offline focus groups were made with a digital recorder, and transcribed verbatim [26]. For the thematic analysis [27] of all focus groups, ATLAS. ti was used. Each author independently read the transcripts to obtain a general impression. Coding was carried out in two cycles; first sentences were assigned detailed codes, and second, codes were merged into subthemes and finally into main themes [28]. The first and last author discussed the codes and (sub)themes. Consensus was reached by reviewing, integrating and modifying the themes and subthemes. All focus groups were conducted and analyzed in Dutch. To present the results, themes and quotes were translated to English. The manuscript was compiled by RO, in consultation with BM and VT, adhering to the Standards for Reporting Qualitative Research (SRQR) guidelines [29].

### 2.2 Ethics

The Social Sciences Ethics Committee of Wageningen University approved the study, certifying that it complied with the Netherlands Code of Conduct for Research Integrity. Prior to the focus groups, participants were provided with an explanation of the study's aim, its voluntary and confidential nature, and were then asked to sign the Informed Consent form. Anonymity was ensured. Transcripts were coded by removing all possible identification of participants. Only the authors had access to the files.

### 2.3 Topic guide construction

The focus group discussions were moderated using a topic guide that was based on relevant theory and literature. The topic guide helped to focus the discussion on the factors involved in decision-making, while offering participants room for sharing their beliefs, needs, and experiences regarding the OCP, and responding to each other. The topic guide began with asking participants about current contraceptive methods they were using, followed up by general experiences, effects, and side-effects. Specific attention was paid to differentiating between knowledge and beliefs about effects and side effects, as well as positive and negative experiences and feelings. The next topic explored how participants made the decision to continue or discontinue with the OCP, including the role of awareness of side effects; and the extent to which the decision was deliberate and conscious. Thirdly, the topic guide delved into who influenced the decision to use the OCP or not, and how participants perceived important others’ behaviors and evaluations regarding (not) using the OCP. The final topic explored whether and how participants discussed effects, concerns and decision-making with others.

## 3 Results

### 3.1 Focus groups

During the focus groups, participants displayed a sense of openness and interest. Personal life stories, secrets and vulnerable topics were addressed on their own initiative. Participants indicated that participation in the focus group was a positive experience. Although participants indicated that they did discuss this topic with e.g. their peers, in-depth discussion and the opportunity to share experiences within the focus groups was felt to be valuable. The topic guide helped capture the variety of experiences expressed by participants in their decision-making process regarding the (dis)continuation of the OCP. Our analysis revealed that these experiences could be represented by several themes and subthemes. The main themes were ‘effects’, ‘side-effects’, ‘towards alternatives’ ‘interpersonal communication with peers’, ‘interpersonal communication with general practitioner’, ‘online information seeking’ and ‘feeling responsible’. Detailed information on all the themes and subthemes, along with illustrative quotes can, be found in Table 2.

**Table 2:** The themes, subthemes and illustrative quotes.

Themes	Subtheme	Illustrative quote
Effects	Cycle control	“I can decide when my period starts.” Woman aged 21, focus group 1
		“I find it and advantage that I can decide for myself when to continue taking the pill or schedule a stop week.” Woman aged 23, focus group 1
	Pregnancy prevention	“It's not that I'm super conscious … This outweighs this. But not getting pregnant is very important to me and that carries more weight.” *Woman aged 23, focus group 4*
	Menstrual pain	“My period was always very heavy. I experienced so much pain that I often couldn't even get out of bed. I did not start using the pill as a contraception, but because it would reduce the symptoms and that's indeed the case.” *Woman aged 21, focus group 3*
	Acne	“A lot of girls start because they say that they have pustules. I had eight classmates, I think, who did that. I used to have quite a lot of pustules myself and I thought hey well, that's a win, I must have this too. Then I went and got it too.” *Woman aged 21, focus group 2*
	Concerns	“I am doubting about starting to use a condom, because I sometimes hear that the chance of getting breast cancer is higher [when using a OCP, e.d.]” Woman aged 19, focus group
Side effects	Awareness of physical side effects	‘‘I thought I was sick because of the continuous bleeding. Think of 3 times a week at least every week. My option was a different contraception and therefore I use the IUD now.” Woman aged 20, focus group 2
	Awareness of side effects	“Yeah, I had less energy, many mood swings, less happy/enthusiastic, psychological side effects, suffered from migraines later on and the pill also affected my libido.” *Woman aged 21, focus group 1*
		“I can imagine that if you like the pill, you continue with using. Or if you start at a young age with using the pill, the side effects sort of creep in and you don't realise it, or consider it normal.” Woman aged 23, focus group 1
Towards alternatives		“And now I have a boyfriend for 2 years. We both hate condoms and I don't want alternative contraception options, so the only thing left is the pill.” Woman aged 21, focus group 3
Interpersonal communication with peers	Starting the pill is normal	“My female friend told me about it once and I started trying too.“*Woman aged 19, focus group 3*
	Discussing side effects	“Yes, I used to accept it and not think about it, but now that you're much more involved … I often talk about it with housemates who have deliberately stopped or … You know, now, I notice now, I don't know if it's our age or now that it's this time or something, that people start to think more or something … Haha. But now I do think it's really too crazy for words that everyone just goes on the pill en masse and …. yeah.” Woman aged 23, focus group 2
Interpersonal communication with the general practitioner	Prescribing the pill is normal	"I think at a young age, when girls have complaints, it is easily advised to take the pill. That it's some kind of standard solution.” *Woman aged 19, focus group 2*
		“This was the same with me! Abdominal pain? Take the pill.” Woman aged 20, focus group 2
Online information seeking		“I watch clips by beautygloss (beautyinfluencer, red.), for example, who has a lot of good tips on anti-conception.” Woman aged 18, focus group 3
Feeling responsible		“I made a deliberate decision to use only the pill. If it goes wrong and you get pregnant, that is your responsibility coming with the choices that you made.” *Woman aged 21, focus group 3*
		“You do feel responsible. At least I was talking to male roommates about it yesterday. And they said “You just stop if you don't like it” … Then I said, but we have to make sure there aren't a hundred thousand children in the world.” Woman aged 21, focus group 4

#### 3.1.1 Effects and interpersonal communication mark initiating the OCP

The majority of the participants started at a young age with using the OCP because of its desired effects. Reducing menstrual pain, managing acne, cycle control and pregnancy prevention were the most frequently mentioned reasons to start using the OCP. Interestingly, participants indicated that they often started using the OCP before they were sexually active. In addition, most of the participants indicated that starting with the OCP was driven by social influences, particularly through interpersonal communication with peers, parents or GP.

#### 3.1.2 Side effects and information seeking

The theme ‘side effects’ included the subthemes ‘awareness of physical side effects’ and ‘awareness of psychological side effects’. Participants mentioned various physical side effects such as acne, low energy, weight gain, decreased libido, more intense menstruation or withdrawal bleed, irregular bleedings, nausea, abdominal pain, back pain, headaches, and varicose veins. Additionally, they reported psychological side effects including feeling emotionally flattened, lonely, more emotional, sad or depressed, experiencing mood swings, and having a short temper. This process of gradually becoming aware of side effects over time often coincided with a search for knowledge, notably with discussing side effects with peers and looking online for information, experiences and advice, again highlighting the influence of social influences through communication.

#### 3.1.3 Towards alternatives

Women who did attribute complaints to the OCP were more likely to discontinue usage. Most women who did choose to discontinue the OCP experienced multiple negative side effects simultaneously, with irregular bleedings being strongly associated with discontinuation. However, women experienced various degrees of uncertainty about the extent to which physical or psychological complaints could be attributed to the OCP. Higher educated women more frequently expressed uncertainty about recognizing side effects and attributing them to OCP use, possibly indicating that they may not recognize side effects as such or questioned whether they could attribute these to the OCP. Conversely, women in the lower educated groups were generally confident in their ability to identify OCP side effects and convinced they would recognize OCP side effects. When women begin to reassess their reliance on the OCP, it frequently triggers the process of exploration of alternative contraception, like an Intrauterine device (IUD) or condoms.

#### 3.1.4 Feeling responsible

During the reconsideration process, the feeling of responsibility for pregnancy prevention strongly influenced the decision outcome. This sense of responsibility may interfere with the desire to discontinue OCP usage. Interestingly, responsibility seems a newly identified theme in the decision-making process. Particularly in the lower educated focus groups (see Table 2) responsibility for pregnancy prevention was of importance, sometimes resulting in the use of two different kinds of anticonception (condoms and OCP).

## 4 Discussion

### 4.1 Discussion of the results

This study set out with the aim of exploring reasons for (dis)continuation of the OCP among Dutch women. The main themes that were identified were effects, side effects, towards alternatives, interpersonal communication with peers, interpersonal communication with the general practitioner, online information seeking and feeling responsible. There are similarities between the results and those of Simmons et al. (2023) [13]. Simmons et al. (2023) assessed the contraceptive journey of participants using interviews and did not specifically focus on OCP use, but mainly on a person's contraceptive journey, and the four phases thereof. Of these four phases (identification of need, method initiation, method use and method discontinuation), the experiences from the participants of the focus groups were primarily related to three phases; method initiation (see themes and (sub)themes discussed in paragraph 3.1.1.), experiences with a method during use and cessation and decisions to stop (see paragraph 3.1.2.). The results of the focus groups provide more insight in the themes and subthemes that play a role in these two last phases, particularly regarding OCP use.

Women indicated that after starting the OCP, continuing its use did not really feel like a choice; instead ‘it [continuing OCP use] feels self-evident’. However, over time, women may experience, and learn to attribute, side effects to the OCP. This may lead women to reconsider using the OCP. Women were more likely to discontinue OCP use when they attributed their complaints to OCP use and when they experienced multiple side effects simultaneously. This finding aligns with the study of Rosenberg et al. [30] stating that a single side effect increases the likelihood of discontinuation with 50%, two by 220% and three by 320%. Furthermore, Wigginton et al. [31] showed that changes in contraception can also occur for beneficial non-contraceptive reasons, like improving acne.

While the current study indicates that attributing complaints to OCP use influences discontinuation, research on the causal attribution of complaints is limited. To our knowledge, only the quantitative study by Westhoff et al. [32] examines attribution of side effects to oral contraception, specifically among a predominant Hispanic and Black sample. While they report that attribution indeed affects the decision making process, we believe it is relevant to further investigate when and how women attribute side effects to OCP use.

Reconsideration was found to be negatively related to feeling responsible for pregnancy prevention; women who felt a strong sense of responsibility were less inclined to reconsider the use of the OCP because of its effectiveness to preventing and planning pregnancy. While the concept of responsibility in relation to contraception is not new [33], responsibility is often examined as a separate and independent factor. Our findings suggest that responsibility is embedded within the decision-making process by serving as a background or foundational aspect that influences how women perceive side effects and consider alternative contraceptive methods. Further work is required to investigate how responsibility affects the decision to (dis)continue the OCP and understand the underlying mechanisms involved.

Finally, social influences, through communication with peers and the general practitioner, influence decision-making. This is also reported by Melo et al. [17], stressing the importance of peers and healthcare providers in different stages of the decision-making process and reflected in the systematic review of Ti et al. [23] in which is shown that the social context affect contraceptive values and preferences. Moreover, the importance of online information seeking in the context of social influences became clear in this study.

The focus groups consisted of women with different (educational) backgrounds, which was reflected in feeling responsibility and experiential attitude, especially when it comes to attributing complaints and side effects to the OCP. However, with a small sample size, these results should be interpreted with caution and further research into differences in decision-making driven by educational level will need to be undertaken.

### 4.2 Considerations

Although the study sample is limited, it included young women of different levels of education, representing a wide audience. Due to Covid-19 restrictions, 3 out of 4 focus groups had to take place online, using WhatsApp. Nevertheless, participants actively replied on each other on a level that was comparable to the offline focus group. Theory and literature was used to create a topic guide that covered beliefs, experiences and communication with important others, while leaving ample room for an open exploration of the topic.

Based on the findings and limitations of this study, several suggestions for further research can be made. Firstly, expanding the sample size and diversifying the participants in terms of age, socioeconomic backgrounds, and cultural diversity could provide a more comprehensive understanding of the experiences and perspectives related to contraception. Furthermore, further exploration of specific themes and subthemes would enhance the depth of analysis. This could involve conducting in-depth interviews or using other qualitative methods that facilitate research into the individual experiences and decision-making processes. And lastly, it would be beneficial to examine how providing balanced information about contraception over an extended period of time influences decision-making and actual usage. Conducting long-term studies could help understand whether comprehensive information leads to better satisfaction with contraception, increased adherence, and improved outcomes.

### 4.3 Practical implications

Results of this study, within the Dutch context, underscore the importance of providing balanced and comprehensive information about the OCP and alternative contraception methods, specifically concerning their effects and side effects. This information dissemination can be effectively executed during contraception consultations, conducted by general practitioners or other healthcare providers, such as practice nurses. Additionally, it is of importance to provide reliable and up-to-date information on contraception on sources that are trusted by the Dutch population. Furthermore, effective communication concerning contraception should acknowledge and address the large individual variability in experienced side effects, helping women to make accurate attributions of possible side effects to the OCP. This can be achieved by providing detailed information on potential side effects and encouraging women to discuss any adverse effects. By adopting this approach, women in the Netherlands can make informed decisions regarding contraception that meets their expectations of safety, reliability and satisfaction. In the Netherlands, the healthcare system prioritises and switches to person-centred care, wherein individual preferences and needs are central. A second implication necessity to promote a more equal distribution of contraception responsibilities between men and women. While contraception is often perceived as primarily the woman's responsibility, this study shows the need for equal involvement of both men and women in contraception decision-making and usage. Encouraging a balanced share of contraception responsibilities can lead to shared decision making in relation to contraception use within relationships.

## 5 Conclusion

The purpose of the current study was to investigate OCP decision-making in the Dutch context, by capturing women's own experiences in order to explore how women decide to (dis)continue OCP use. Overall, this study shows that the main themes that are part of the decision-making process are effects, side effects, towards alternatives for contraception, interpersonal communication with peers, interpersonal communication with the general practitioner, online information seeking and feeling responsible. Especially the decision to start using the OCP is highly facilitated by normalization through peers and the Dutch health care system, driven by desired effects such as pregnancy prevention, cycle control or curing acne. However, over time, part of the women become aware of physical side effects, such as bleeding, and/or psychological side effects such as mood swings. Awareness of side effects can be heightened through discussions with peers and online information seeking. Nonetheless, feeling responsible for pregnancy prevention may reduce some women's inclination to consider alternatives. These results imply that supporting Dutch women in contraception decision-making can be improved in at least two ways: first, by providing balanced information about the OCP and alternative contraception's, addressing both effects and side effects. Second, by emphasizing and normalizing the shared responsibility for contraception of both man and women.

## Ethics statement

This study complies with the ethical standards outlined by the Social Sciences Ethics Committee of 10.13039/501100004890Wageningen University and Research, approval number 2020-51-Thissen. Prior to the start of the focus groups, informed consent was obtained from all participants.

## Author contribution statement

Rebecca S. Otte: Conceived and designed the experiments; Analyzed and interpreted the data; Contributed reagents, materials, analysis tools or data; Wrote the paper. Bob C. Mulder: Analyzed and interpreted the data; Conceived and designed the experiments; Contributed reagents, materials, analysis tools or data. Vera T.M. Thissen: Conceived and designed the experiments; Performed the experiments; Contributed reagents, materials, analysis tools or data.

## Data availability statement

Data will be made available on request.

## Declaration of competing interest

The authors declare that they have no known competing financial interests or personal relationships that could have appeared to influence the work reported in this paper.
